# Endogenous Controls for the Evaluation of Osteoarthritis-Related miRNAs in Extracellular Vesicles from Bone-Marrow-Derived Mesenchymal Stromal Cells and the Impact of Osteoarthritis Synovial Fluid

**DOI:** 10.3390/biom12020316

**Published:** 2022-02-16

**Authors:** Enrico Ragni, Carlotta Perucca Orfei, Marco Viganò, Federico Valli, Laura de Girolamo

**Affiliations:** 1 Laboratorio di Biotecnologie Applicate all’Ortopedia, IRCCS Istituto Ortopedico Galeazzi, Via R. Galeazzi 4, I-20161 Milan, Italy; enrico.ragni@grupposandonato.it (E.R.); carlotta.perucca@grupposandonato.it (C.P.O.); marco.vigano@grupposandonato.it (M.V.); 2 Chirurgia Articolare Sostitutiva e Chirurgia Ortopedica (CASCO), IRCCS Istituto Ortopedico Galeazzi, Via R. Galeazzi 4, I-20161 Milan, Italy; mail@federicovalli.it

**Keywords:** bone marrow, mesenchymal stromal cells, extracellular vesicles, osteoarthritis, synovial fluid, miRNAs, reference genes

## Abstract

Bone-marrow-derived stromal cells (BMSCs) have emerged as promising therapeutic option for the treatment of osteoarthritis (OA) due to their tissue regenerative and anti-inflammatory features. BMSCs’ clinical potential is mainly ascribed to their released factors and extracellular vesicles (EVs), whose therapeutic portfolio may be modulated by the environment in vivo or specific priming in vitro. Within the array of molecules shaping EVs’ power, miRNAs are considered privileged players. In this frame, a correct EV-miRNA detection and quantification is mandatory to understand and possibly boost BMSCs potential, either when envisioned as cell therapeutics or when proposed as producer of cell-free and clinical grade EVs. The aim of this study is to identify reliable reference genes (RGs) to study miRNAs in BMSC-EVs cultivated under standard or OA synovial fluid (OA-SF). miR-23a-3p and miR-221-3p emerged as the best candidates, respectively. Moreover, when both conditions were analyzed together, miR-24-3p resulted the most stable RGs, allowing for a sharper comparison of EVs content, further validated on the OA-related miRNA-193b-5p. The different RG stability ranking depending on the culturing conditions, as well as its divergence with respect to adipose (ASCs) and amniotic (hAMSCs) MSCs, confirm that miRNA RG selection in EVs is a mandatory step and that the identification of the most reliable candidate is greatly depending on the cell type and culturing/environmental conditions.

## 1. Introduction

Osteoarthritis (OA) affects more than 600 million individuals worldwide [1]. OA etiology is multifactorial and includes aging and obesity as well as joint injury [2]. Knee OA accounts for almost 4/5 of OA burden [3]. OA is a chronic structural disease developing over as long period and major OA clinical symptoms include chronic pain, joint instability, stiffness and joint space narrowing [4]. OA is a chronic structural disease developing over a long period of time and is characterized by different phases. In the early stages, the cartilage becomes worn and torn, as well as inflamed, and this process leads to hardened cartilage that makes moving the surrounding joint more difficult. In mild stage, X-rays show larger bone spurs, but cartilage is still thick enough to prevent friction between bones. Eventually, in late stages, bones come in contact with each other, causing pain, loss of movement and function. Because the molecular mechanisms shaping OA initiation and progression are poorly understood, effective pharmacological interventions to slow down disease progression and restore cartilage damage are missing, and arthroplasty is often the only effective treatment at an advanced stage of the disease [5]. For these reasons, clinicians and researchers are actively tuning their focus to identify new treatments able to delay surgical interventions and the need for joint replacement.

In this frame, in recent years disease-modifying strategies, including biological approaches [6], have gained interest [7]. These approaches aim to alleviate OA structural damages and modify the underlying OA pathophysiology to prevent long-term disability. Two strategies are currently under investigation. Disease-modifying OA drugs targets inflammatory cytokines, matrix-degrading enzymes and the Wnt pathway to modulate the degenerative changes in osteoarthritic cartilage. Regenerative approaches aim to counterbalance the loss of cartilage matrix by stimulating chondrogenesis in endogenous stem cells and matrix anabolism in chondrocytes. These approaches can be particularly effective in treating patients in the early stage of OA when significant cartilage remains, rather than in the late stage of OA. In the basket of new options under investigation, adult mesenchymal stromal cells (MSCs), either freshly isolated or expanded, emerged among the most innovative tools for OA treatment, due to tissue regenerative [8] and anti-inflammatory [9] properties, as reported in pioneeristic trials showing, together with safety, improved pain levels and function [10] and regenerated hyaline articular cartilage thickness at 6 months [11,12]. MSCs may be isolated from several tissue sources [13], with bone marrow being a historically privileged option. Consistently, both bone marrow aspirate and bone marrow MSCs (BMSCs) expanded under good manufacturing practice (GMP) conditions [14,15] have been and are still under clinical investigations, showing promising results in particular for safety and pain reduction through modulation of inflammation. In addition, early clinical data suggests BMSCs and BMSCs-enriched products may help stimulate a more robust hyaline cartilage repair tissue response [16]. Of note, both anti-inflammatory and tissue regenerative effects are mediated by an active paracrine signaling. In fact, recently, MSCs have been termed as Medicinal Signaling Cells since these cells secrete bioactive factors that are immunomodulatory and trophic [17].

Together with the most characterized secreted soluble molecules, such as cytokines and chemokines, an array of bioactive factors including lipids and nucleic acids, as miRNAs, are shuttled as embedded within extracellular vesicles (EVs) [18]. In particular, miRNAs, mirroring their role for disease development or management, resulted the most important players framing EVs potential for several therapeutic settings [19]. Regarding OA, to date, the role of miRNAs in shaping the disease and, by consequence, in defining their potential as therapeutic players when exogenously administered (e.g., by EVs) is under active investigation [20], and although available in vitro/in vivo results allow for positive evaluation of these molecules, caution has to be taken until more data will be available [21]. In this context, a fundamental pillar to envision EVs as disease-specific therapeutics is the definition of reliable and easy methods to both determine the presence and quantify the amount of specific miRNAs, a possible limitation of EV-based therapy. In fact, in MSC-EVs, even for the most abundant miRNA, no more than one copy per vesicle is present [22], a minimal ratio of 100 MSC-EVs per target cell is needed to allow transfer of abundant miRNAs [23], and in several cell types, including chondrocytes, only a few thousand MSC-EVs can be incorporated in a day [24]. Therefore, only abundant miRNAs may be considered as potentially active and their reliable quantification for therapeutic effect prediction crucial. In addition, cell behavior and by consequence EV composition is affected by many factors including cell culture parameters in vitro or environmental conditions in vivo, which introduce the difficulty for EV characterization and comparison under different settings. As an example, in adipose-derived MSCs (ASCs) in vitro inflammatory priming often used to mimic OA conditions was able to modulate EV-miRNA fingerprint [25], as similarly observed after treatment with OA synovial fluid that is more effectively recapitulating in vivo conditions [26]. Eventually, easy and reliable strategies to quantify EVs content might allow to evaluate the impact of detrimental or unwanted effects [27], such as differential recovery due to alternative isolation techniques, degradation due to prolonged or improper storage and the presence of potentially harmful molecules as pathogenic miRNAs [28] or proteins [29] as seen for neurologic disorders.

Accounting for the differences arising from either cell source or inter-donor or treatment variability or combination of these factors, reference genes (RGs) with stable expression are crucial, although, to date, no recognized RGs are generally accepted to normalize EV-miRNA expression. Therefore, in this work, we sifted a panel of putative miRNA RGs in EVs released from BMSCs obtained under conventional culturing conditions or treatment with OA synovial fluid.

## 2. Materials and Methods

### 2.1. OA-Synovial Fluid Collection

Synovial fluid (SF) was collected from 13 OA (mean age: 69 ± 8; 8 females and 5 males; Kellgren and Lawrence III–IV grade) patients who underwent knee arthroplasty. The OA-SF was collected by puncture before arthroplasty. OA-SF were centrifuged at 16,000× *g*, 10 min at RT, to remove debris, and the supernatants were stored at −80 °C. Before experiments were performed, single aliquots were pooled.

### 2.2. OA-SF Characterization

A total of 250 μL OA-SF were supplemented with 12.5 μL of 40 mg/mL Hyaluronidase (Sigma-Aldrich, Milan, Italy) and 1.25 μL of Protease Inhibitor Cocktail (abcam, Cambridge, MA, USA). Incubation was performed for 30 min at 37 °C. Two-fold diluted digested OA-SF was used for cytokine (IL-1β, IL-6, IL-8, IFNγ and TNFα) detection with the RayBio^®^ Human ELISA Kit (RayBiotech, Norcross, GA, USA), following manufacturer’s protocol and four technical replicates. Concentrations were determined by comparison with standard samples.

### 2.3. BMSCs Isolation and Expansion

Total bone marrow aspirate from three female donors (mean age: 50 ± 2) was seeded in αMEM (Thermo Fisher Scientific, Waltham, MA, USA) supplemented with 10% fetal bovine serum (FBS) (Thermo Fisher Scientific, Waltham, MA, USA) at the concentration of 50,000 total nucleated cells/cm^2^. Cells were cultured at 37 °C, 5% CO_2_, and 95% humidity. After 3 days, the supernatant was discarded and replaced by fresh complete medium. After 2 weeks, colonies of BMSCs were detached and cells seeded in the same culture conditions. BMSCs were used at passage three.

### 2.4. BMSCs Characterization by Flow Cytometry

Flow cytometry scored the presence or absence of MSC (CD90-FITC clone REA897, CD73-PE clone REA804, CD105-PerCP Vio700 clone REA794, CD44-PE Vio770 clone REA690, CD271-PE clone REA844) or hemato/endothelial (CD34-FITC clone AC136, CD31-PerCP Vio700 clone REA730, CD45-PE Vio770 clone REA747) markers (Miltenyi Biotec, Bergisch Gladbach, Germany) with a CytoFLEX flow cytometer (Beckman Coulter, Fullerton, CA, USA) collecting a minimum of 30,000 events [30]. Antibodies were used in the following combinations: CD73/90/105/44 and CD34/271/31/45.

### 2.5. BMSC-EVs Isolation and Characterization

When at 70% confluence, BMSCs were cultured for 48 h in either standard complete medium or medium supplemented with 50% pooled OA-SF. BMSCs were then washed three times with PBS, and αMEM medium without supplement added (12 mL per T175 cell culture flask). After 48 h, culture supernatant was collected and serially centrifuged (Beckman Coulter, Fullerton, CA, USA) at 4 °C to eliminate debris and floating cells at 376× *g* for 15 min, 1000× *g* for 15 min, 2000× *g* for 15 min and twice at 4000× *g* for 15 min each to remove apoptotic bodies. Conditioned medium was divided and processed as follows:

Flow cytometry: Conditioned medium was 1:1 diluted with PBS and one aliquot was left unstained, one aliquot supplemented with carboxyfluorescein succinimidyl ester (CFSE) (Sigma-Aldrich, Milan, Italy) (1 µM final concentration) and incubated for 30 min at 37 °C and one aliquot, after CFSE supplementation, treated with one of the following antibodies (CD9-APC clone HI9A, CD63-APC clone H5C6, CD81-APC clone 5A6, CD44-APC clone BJ18, CD73-APC clone AD2, CD90-APC clone 5E10; Biolegend, San Diego, CA, USA) following manufacturers’ instruction. After a further 1:3 dilution with PBS, samples were analyzed with a CytoFlex flow cytometer comparing outcomes with those obtained running FITC-fluorescent beads of 160, 200, 240, and 500 nm (Biocytex, Marseille, France). At least 30,000 events were collected.

Nanoparticle tracking analysis (NTA): Conditioned medium was 1:1 diluted in PBS and visualized by Nanosight LM10-HS system (NanoSight Ltd., Amesbury, UK). Five recordings of 60 s were performed for each sample. Network traffic analysis (NTA) software v3.4 (NanoSight Ltd., Amesbury, UK) analyzed collected data, providing both concentration measurements and high-resolution particle size distribution profiles.

### 2.6. Total RNA Isolation and miRNA Profiling

Conditioned medium was 1:1 diluted in PBS and ultracentrifuged (Beckman Coulter, Fullerton, CA, USA) at 100,000× *g* for 9 h at 4 °C, and pellet-processed with miRNeasy and RNeasy Cleanup Kits (Qiagen, Hilden, Germany). To evaluate RNA recovery and cDNA synthesis efficacy, samples were spiked in with exogenous *Arabidopsis thaliana* ath-miR-159a (30 pg) synthetic miRNA before RNA extraction. cDNA for each sample was prepared as previously reported [31]. miRNA expression analysis was performed with the OpenArray system (Life Technologies, Foster City, CA, USA) into 384-well OpenArray plates according to the manufacturer’s instructions. Missing values and values with C_RT_ > 27 were set equal to the detection limit of C_RT_ = 28 as per manufacturer’s specification, in order to avoid results that may be stochastic. C_RT_ = 28 value, roughly corresponding to 1 target copy with OpenArray technology and protocol, was due to the need for preamplification reaction and the low volume of the reaction. When in two out of three samples values were C_RT_ > 27, these samples were considered as not present. Each specific candidate was considered for analysis only when present in all 3 EV isolates. Then, ath-miR-159 spike-in C_RT_ were used for the equalization of technical differences during the whole process and the mean of positive candidates in all samples was used to equalize subtle differences in the amount of starting RNA.

According to the literature, 14 miRNAs and 1 small RNA (U6 snRNA) were selected for stability analysis and optimal reference gene (RG) identification (Table 1) [30,32,33,34,35,36,37,38,39,40,41,42,43]: has-let-7d-5p 002283; hsa-miR-16a-5p 000391; has-miR-22-5p 002301; hsa-miR-23a-3p 000399; has-miR-24-3p 000402; hsa-miR-26a-5p 000405; has-miR-29a-5p 002447; hsa-miR-34a-5p 000426; hsa-miR-101-3p 002253; hsa-miR-103a-3p 000439; hsa-miR-221-3p 000524; hsa-miR-423-5p 002340; hsa-miR-425-5p 001516; has-miR-660-5p 001515; U6 snRNA 001973. The following assay was selected for OA-related miRNA analysis (Table 1) [44]: has-miR-193b-5p 002366.

### 2.7. Data Analysis

Putative RG stability was calculated with four applets (geNorm [45], NormFinder [46], BestKeeper [47] and ΔC_t_ method [48]). The four algorithms are included in the RefFinder web-based comprehensive tool (https://www.heartcure.com.au/reffinder/) (accessed on 6 December 2021) [49] that, based on the rankings from each algorithm, assigns an appropriate weight to an individual candidate and calculates the geometric mean of their weights, eventually resulting in an overall final ranking.

Principal components analysis (PCA) plots were generated with ClustVis package (https://biit.cs.ut.ee/clustvis/) (accessed on 3 December 2021) [50] with the following settings: no transformation and no scaling.

### 2.8. Statistical Analysis

Statistical analyses were performed using GraphPad Prism Software (GraphPad, San Diego, CA, USA). Grubb’s test was used to identify and exclude possible outliers. One-sample t-test with hypothetical mean value of 1 (*n* = 3) was performed to evaluate miR-193b-5p modulation. Significance level was set at *p*-value ≤ 0.05.

## 3. Results

### 3.1. BMSCs and EVs Characterization

**Figure 1:**
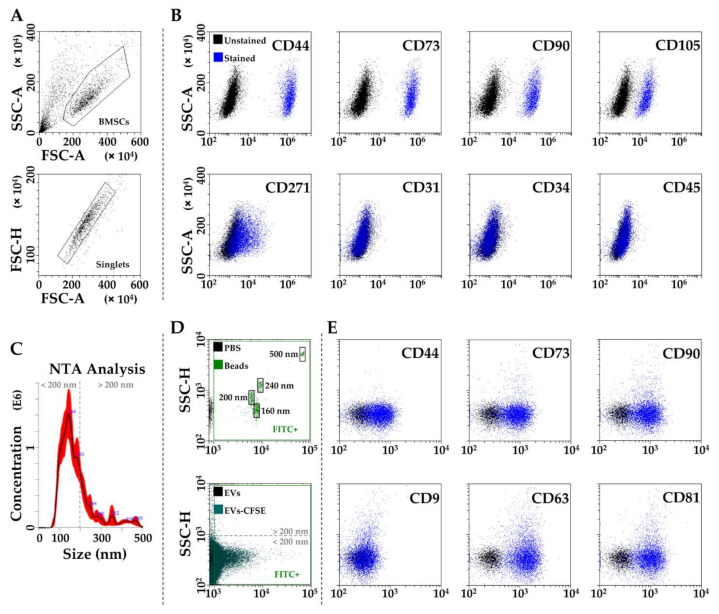
Characterization of BMSCs and BMSC-EVs. (**A**) After flow cytometry BMSCs identification via physical parameters and singlets selection, (**B**) analysis of general MSC (CD44, CD73, CD90 and CD105), adult MSC (CD271) and hemato-endothelial (CD31, CD34 and CD45) markers, confirms BMSCs identity. In panel (**B**), representative plots of FITC (CD34/90), PE (CD73/271), PC5 (CD31/105) and PC7 (CD44/45) channels for events gated under “Singlets” in panel (**A**) are shown. (**C**) Representative NTA analysis of BMSC-EVs. (**D**) Flow cytometry of FITC-labeled nanoparticles assuring calibration of flow cytometer and comparison with CFSE-labeled BMSC-EVs. (**E**) Presence of MSC-markers CD44, CD73 and CD90 and EV-markers CD9, CD63 and CD81 on CFSE-labeled BMSC-EVs. In panel (**E**), representative plots of APC channel for all tested antibodies for events gated under FITC^+^ gate of EVs + CFSE in panel (**D**) are shown.

MSC cell-surface antigens [51], CD44, CD73, CD90 and CD105, were highly expressed. CD271, a distinctive adult-MSC marker [52] was also detected. On contrary, hemato-endothelial markers [51], such as CD31, CD34 and CD45, were negative (Figure 1A,B).

By NTA, BMSC-EVs were within the expected extracellular vesicle size range (mode of 132 ± 7 nm) (Figure 1C), supporting what previously reported for this EV type [53]. Flow cytometry confirmed BMSC-EVs dimensional range by direct comparison with nanobeads of defined size (160, 200, 240 and 500 nm) (Figure 1D). CD44, CD73 and CD90, all defining BMSC-EVs [54] and present on parental cells, were strongly positive (Figure 1D). As in MSC-EVs from adipose tissue [41], amniotic membrane [41], cord blood [53] and previously shown in bone marrow as well [53], BMSC-EVs strongly expressed both CD63 and CD81 EV markers, while CD9 staining gave an extremely weak signal (Figure 1E).

### 3.2. Expression of Candidate Reference Genes

**Figure 2:**
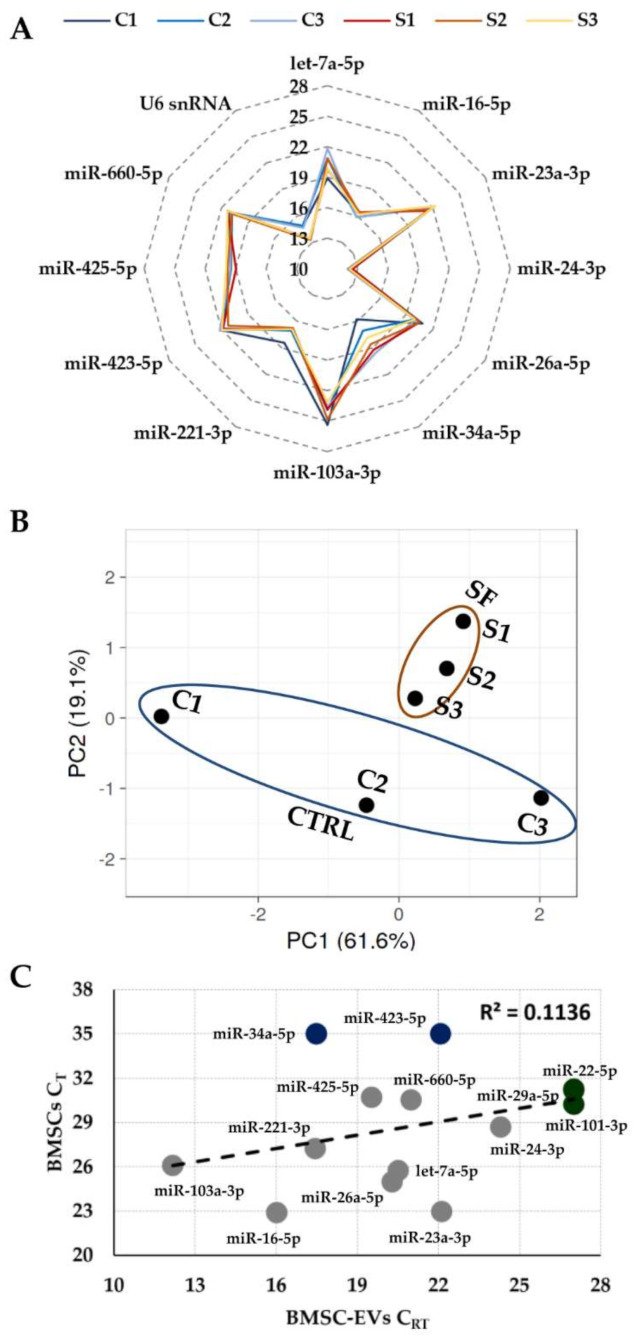
Expression of candidate RGs in BMSC-EVs. (**A**) The graph shows the C_RT_ values of the 12 detected RGs in both standard (C1 to 3) and OA-SF treated (S1 to 3) BMSC-EVs samples. C_RT_ values have a 10 to 28 range as indicated. (**B**) Principal Component Analysis of all C_RT_ data from detected RGs of standard (C1 to 3) and OA-SF treated (S1 to 3) BMSC-EVs samples. No transformation and scaling were applied. First two axes shown cover 80.7% of the variation in composition. (**C**) Regression analysis of all candidate RGs in BMSC-EVs (C_RT_ from this study) and BMSCs (C_T_ from [56]). The R^2^ correlation coefficient is shown. In grey, RGs expressed in both datasets, in blue only those expressed in BMSC-EVs and in green only those expressed in BMSCs.

The presence of the 15 selected RGs (Table 1) was assessed in purified BMSC-EVs (Table S1). miR-24-3p had the highest expression, whereas miR-103a-3p had the lowest (Figure 2A). miR-22-5p, miR-29a-5p and miR-101-3p were not considered for analysis since they were not detected in all donors. SF treatment did not lead to their expression, but allowed to separate samples into two subgroups, as emerged with PCA (Figure 2B), although correlation analysis revealed high R^2^ values between control and treated samples (0.92 ± 0.05). Contiguity analysis confirmed that none of the tested candidates resided within the same gene cluster, thus minimizing the possibility of including co-regulated miRNAs in the stability analysis [55].

Notably, lack of amplification for miR-22-5p, miR-29a-5p and miR-101-3p confirmed the absence of cellular contamination, since in a previous publication on cellular BMSC-miRNAs scored by qRT-PCR they were reported as positively amplified in a fashion similar to, e.g., miR-425-5p and miR-660-5p [56]. Further, the detection of miR-34a-5p and miR-423-5p in EVs, that resulted absent in cellular BMSC miRNome, suggested a preferential EV incapsulation. This was also corroborated by a regression analysis between EV and cellular BMSC-miRNAs from our and published dataset that resulted with a very low correlation coefficient (R^2^ of 0.11) (Figure 2C).

### 3.3. RGs Stability Analysis

As a first step, four algorithms were run to assess putative RG stability in BMSC-EVs (Table 2A). Genorm identified the couple miR-23a-3p/miR-425-5p (M value of 0.074) as the most stable candidates. miR-34a-5p ranked in the last position (0.940). NormFinder identified again miR-23a-3p and miR-425-5p as the best RGs with a SV of 0.037. miR-34a-5p was confirmed to be the worst performer (2.006). miR-425-5p and miR-23a-3p again ranked in the first positions using BestKeeper, with a SD of 0.05 and 0.07, respectively. miR-34a-5p endorsed its instability (SD of 1.41). Delta Ct method gave an identical outcome for miR-23a-3p, first with SD of 0.64, while in second position appeared miR-16-5p (SD of 0.64). No changes emerged for miR-34a-5p, laying in the bottom of the ranking. Eventually, to identify a definitive and reliable hierarchy, an integration and normalization of the data was performed. The geometric mean confirmed miR-23a-3p and miR-425-5p as the most stable RGs, and, as expectable, miR-34a-5p as the least stable.

Second, before monitoring the effect of OA synovial fluid on candidate RGs (Table 2B), the amount of few soluble factors was evaluated in the pooled SF. Out of 5 molecules often associated with OA inflammatory status, TNFα was not detected, while IL-1β resulted 98.8, IL-6 209.0, IL-8 56.4 and IFNγ 86.4 pg/mL. Then, in EVs released from OA-SF treated BMSCs, with Genorm, miR-221-3p/U6 snRNA resulted the most stable RGs (M of 0.049 for both), and miR-103a-3p the worst one (0.590). miR-221-3p (SV of 0.025) ranked again first with Normfinder, followed by miR-16-5p (0.063), while miR-103a-3p laid at the bottom of the group (0.831). After BestKeeper processing, miR-221-3p and U6 snRNA confirmed their stability (SD of 0.03 and 0.07, respectively), as miR-103a-3p its instability (0.59). As per Normfinder analysis, with Delta CT algorithm miR-221-3p and miR-16-5p emerged as most stable RGs (SD of 0.41 and 0.42, respectively), whilst miR-103a-3p again last (0.90). Eventually, geometric mean substantiated the first two positions for miR-221-3p and U6 snRNA, followed by miR-16-5p, and the higher instability for miR-103a-3p.

Third, a comprehensive analysis encompassing all samples was conducted, to obtain reliable RGs when directly comparing EVs released from BMSC cultured under either standard or OA-like conditions (Table 2C). miR-24-3p resulted first in the ranking with all the four applets (Genorm M of 0.135, Normfinder SV of 0.213, BestKeeper SD of 0.18 and Delta CT SD of 0.64), and miR-16-5p again first with Genorm (M of 0.135) and second with the other three algorithms (Normfinder SV of 0.263, BestKeeper SD of 0.20 and Delta CT SD of 0.66). miR-34a-5p always laid last (Genorm M of 0.876, Normfinder SV of 1.521, BestKeeper SD of 1.12 and Delta CT SD of 1.60). Therefore, global mean confirmed the ranking with miR-24-3p and miR-16-5p at its top and miR-34a-5p at the bottom.

### 3.4. Impact of RGs Choice on the Quantification of Specific Target miRNAs

**Figure 3:**
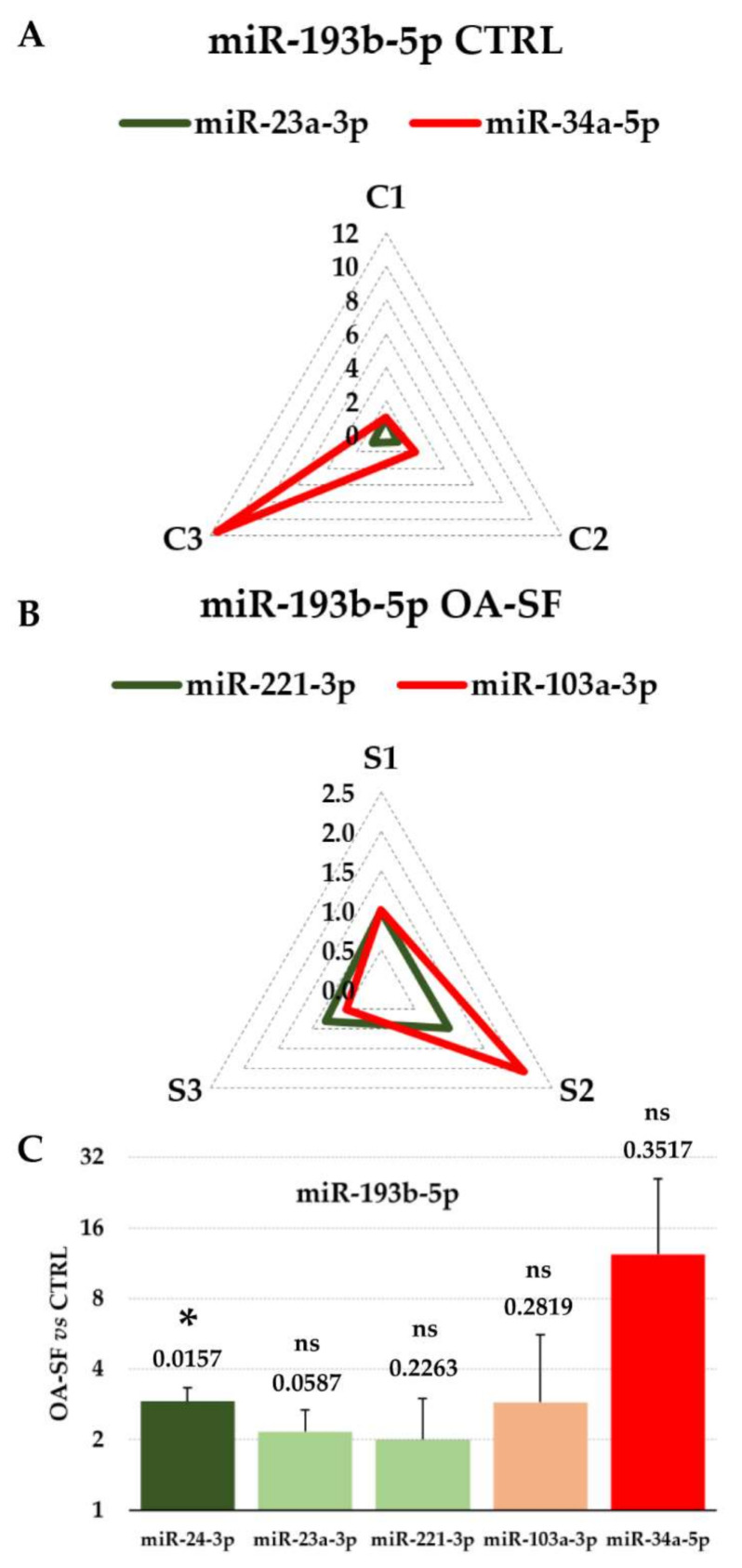
Effects of different RGs on the normalization of OA-related miR-193b-5p expression. (**A**) miR-195b-5p was normalized in standard (C1 to 3) samples with either stable miR-23a-3p or unstable miR-34a-5p using C1 sample as milestone (set as 1) for comparison with C2 and C3. Ratios are indicated (0–12 scale). (**B**) miR-195b-5p was normalized in OA-SF (S1 to 3) samples with either stable miR-221-3p or unstable miR-103a-3p using C1 sample as milestone (set as 1) for comparison with C2 and C3. Ratios are indicated (0–2.5 scale). (**C**) Effects of RG normalization on the modulation of miR-195-5p differential expression between standard (CTRL) and OA-SF treated samples. CTRL samples set as 1 for each pair (mean with standard deviation is shown, *n* = 3); * for *p*-value ≤ 0.05; ns for *p*-value > 0.05; numerical values on top of each bar stand for *p*-value.

The impact of RG choice on specific miRNA evaluation was performed on miR-193b-5p expression, due to its role as OA protective player [44] and, by consequence, as putative miRNA therapeutic to be shuttled via nanoparticles such EVs [57]. First, miR-193b-5p levels in the BMSC-EVs isolates were compared using both reliable (miR-23a-3p) and unreliable (miR-34a-5p) RGs (Table 2A). Notably, when miR-23a-3p was selected for normalization, a closer amount between isolates emerged (0.94 ± 0.09 average value with C1 sample as milestone) with respect to miR-34a-5p that gave misleading conclusions (4.86 ± 5.82) for both averaged and single evaluations (Figure 3A). Similar results were obtained for OA-SF BMSC-EVs, using miR-221-3p as stable and miR-103a-3p as unstable RGs (Table 2B). In the first case, very comparable miR-193b-5p amounts were determined (0.94 ± 0.10), with respect to a greater variability in the second condition (1.20 ± 0.81) where both double and half amounts were detected for C2 and C3 samples, respectively (Figure 3B). Eventually, the ratio between standard and OA-SF treated BMSC-EV samples was checked by means of reliable and unreliable RGs identified in both single and merged conditions (Table 2A–C). As clearly emerged in Figure 3C, only miR-24-3p allowed for the detection of a significant (SF vs. CTRL ≥ 2, *p*-value ≤ 0.05) increase in OA-SF EV samples while, in all the other cases, also when using the best RGs for the single conditions, this criterion was never met, resulting in extremely high fluctuations when using miR-34a-5p.

## 4. Discussion

In this report, an integrated method based on four well-known stability algorithms was proposed to identify reliable miRNA RGs in EVs released from BMSCs. Of note, OA-SF treatment altered miRNA stability and reliable RG selection, confirming the usefulness of this study. Eventually, the use of the most correct RG and the effects of suboptimal RG choice was validated on the differential expression of cartilage protective and putative OA-therapeutic miR-193b-5p.

EVs may be envisioned either as intercellular mediators [58] or as cell-free nanoshuttles [59] to target specific/diseased tissues or organs. In both cases, a precise characterization of both embedded molecules and their amount is mandatory. In this frame, miRNAs are postulated as the most important vehicle of information [60]. Furthermore, miRNA cargo may be altered both naturally, e.g., when secreting cells interact with target tissue or environment in vivo, and artificially, e.g., when cells are cultivated under specific culture conditions in vitro or when EVs are engineered to load specific drugs or miRNAs. In the scenario of OA, all these conditions were reported and actively studied to understand and increase MSCs and derived EVs potential. Our group recently showed how ASCs interaction with OA-SF alters EV-miRNAs cargo, with an increase of chondro-protective signals and reduction of T-cell activation [26]. Similarly, ASC inflammatory priming leaded to a more pronounced cartilage regenerative and anti-inflammatory EV phenotype [25]. Moreover, customized drug-loaded EVs are gradually applied in the field of OA therapy by either loading miRNA into the secreting cells or directly mixing EV with purified particles [61,62,63], although this technology has to be improved in terms of efficiency [64]. Eventually, differential tissue regenerative and inflammatory modulating properties related to EV-miRNAs emerged comparing ASCs and hAMSCs [41]. It should be noted that all these approaches and studies relied on the quantification and comparison of molecules between different donors, tissue sources or conditions. In the majority of cases, the most convenient procedure is miRNA detection by qRT-PCR techniques, with the normalization relying on the global mean of a large number of miRNAs considered as the most sensitive approach [65]. Nevertheless, due to the large amount of RNA needed to score the entire miRNome, or at least part of it, leading to the loss of both a remarkable amount of EVs and time for data interpretation, the use of few RGs emerged as an affordable and convenient technique. The main pitfall of this approach is the lack of universal RGs for EV-miRNAs, and reports on a selected panel of putative RG clearly showed that, at least for MSCs, both source and condition may heavily affect the interpretation.

For the abovementioned reasons, in this report we sifted in BMSC-EVs the putative miRNA RG list previously studied and validated in ASC- and hAMSC-EVs together with other EV types (Table 1). miR-23a-3p and miR-425-5p resulted the best performers (Table 2A). As a further confirmation of source specificity, miR-23a-3p resulted as the best candidate in ASC-EVs [37] but was not detected in hAMSC-EVs [39]. Similarly, miR-425-5p was expressed in ASC-EVs, although not resulting among the most stable RGs, and absent in hAMSC-EVs. These results could suggest miR-23a-3p as good RG for EV-miRNAs from adult MSCs. Nevertheless, under OA-SF treatment, miR-23a-3p lost its stability between donors, resulting in the 8th position (Table 2B). This did not recapitulate the excellent miR-23a-3p performance in ASC-EVs after mild inflammatory treatment mimicking OA condition [37], suggesting that either OA-SF is a more complex scenario than an in vitro designed cytokine cocktail or BMSCs and ASCs respond differently to a similar environmental condition. This paradigm was further emphasized with the poor miR-23a-3p interdonor stability in EVs from ASCs primed with high concentrations of IFNγ [30], proposed to increase MSC anti-inflammatory and tissue-healing properties [66]. The divergent influence of the same condition on different RG candidates or on different MSC types was again emphasized by miR-221-3p that, in an opposite fashion with respect to miR-23a-3p, resulted the best performer both after OA-SF treatment (Table 2B) and after ASCs cultivation in mild inflammatory environment mimicking OA [37]. The optimal U6 snRNA performance was quite surprising (Table 2B). In the previous analysis on ASC- and hAMSC-EVs, even under inflammatory stimuli, it always laid close to or at the bottom of the rankings, supporting the theory of its poor use as miRNA-RG for EVs due to the fact that U6 snRNA biogenesis is mechanistically separated from miRNA biogenesis [67]. In the herein reported results, cytoplasmic contamination interfering with EVs quantification and possibly explaining U6 snRNA performance should not be an explanation. In fact, correlation analysis with published data on cytoplasmic miRNAs detection in BMSCs with the same technique, although for different donors, gave rise to very low correspondence (Figure 2C). On the contrary, this analysis suggests in BMSCs a preferential EV-miRNA encapsulation, as previously reported for other cell types [68], and as similarly happens for mRNAs [69].

Eventually, stable RGs analysis for comparing EV-miRNAs between standard and OA-SF treated BMSCs highlighted miR-24-3p and miR-16-5p as the best candidates, while miR-23a-3p and miR-221-3p performed poorly (Table 2C). This again confirms that for each specific study relying on single or few RGs, a proper validation is necessary, especially when few donors are sifted. This was further emphasized by miR-193b-5p differential expression investigation (Figure 3C). In fact, miR-23a-3p, miR-221-3p and even OA-SF poor miR-103a-3p resulted in a CTRL vs. OA-SF ratio similar to the value obtained with miR-24-3p but without statistical significance. miR-193b-5p is reduced in OA cartilage leading to increased MMP3 and MMP13 and its overexpression in inflamed chondrocytes restores their basal levels [44]. Therefore, miR-193b-5p might be envisioned as a new therapeutic molecule able to prevent cartilage matrix degradation, and a proper detection of its levels in BMSC-EVs from different donors, together with its modulation under specific priming or environmental conditions, is a crucial step for its future use both in clinical grade EVs and in BMSC-containing products. Intriguingly, the identification of miR-24-3p and miR-16-5p as the best performers open the question whether a proper RG in a particular disease setting should or should not have a role in the pathology itself. In fact, if for standard BMSC-EVs-specific miR-23a-3p, no reports associated with OA are published, for the OA-SF-specific miR-221-3p and standard/OA-SF specific miR-24-3p/16-5p, more data are present in the literature. miR-221-3p is downregulated in OA and its upregulation prevents IL-1β-induced ECM degradation in both chondrocytes [70] and synoviocytes [71]. Similarly, miR-24-3p expression level is lower in OA and its overexpression significantly attenuates MMP13 and ADAMTS5 protein expression [72]. Moreover, miR-24-3p favors M2 macrophage anti-inflammatory phenotype [73]. Conversely, miR-16-5p is significantly higher in OA cartilages and induces the expression of matrix metalloproteinases and ADAMTS [74]. Therefore, the issue of using a disease-related, in this case OA-related, miRNA as RG for quantification in EVs for studies on the same disease is debatable. In our opinion, this does not hamper their use for a variety of reasons. First, for many miRNAs data are still missing regarding their function and role in the development of several diseases. Second, the fluctuations observed in the target disease, as for miR-221-3p, miR-24-3p and 16-5p, do not recapitulate possible fluctuations in the EVs under study, even if secreting cells are in the similar conditions of the diseased tissues (e.g., cartilage in OA in vivo and BMSCs in OA-SF in vitro). This is also emphasized from data in Figure 2C, where cellular miRNA levels, possibly altered by environmental and culture conditions, are not reflected with a similar fashion in EVs. Thus, we believe that the crucial requirement for an optimal RG is its stability in the given condition rather than a possible role in the analyzed setting.

The main limitation of this report is the reduced number of donors; nevertheless, we believe that the presented results will be an important milestone for the study of EVs and miRNAs from BMSCs in general and in the OA setting in particular. Future studies aimed at confirming these data in other conditions or with a larger number of donors will be required.

## 5. Conclusions

This study allowed the definition of two distinct albeit connected results. First, EV-miRNA RGs from BMSCs cultivated under standard or OA-like condition were identified, miR-23a-3p and miR-221-3p respectively, with miR-24-3p as the most suitable for investigations aimed at comparing the two conditions. The different outcomes with respect to other MSC types, as ASCs and hAMSCs, highlighted the need of cell type and condition specific analyses for the identification of the most stable EV-miRNA RGs, especially when few donors or miRNAs are scored. Future studies are mandatory to integrate RG stability data to allow the selection, if possible, of few RGs to be used in different settings or sources for EVs, although, at present, there is not such a glimpse of light and pioneeristic available data suggest this result might be not reachable.

## Figures and Tables

**Table 1:** Candidate RGs and OA-related miRNA and their target sequences.

Accession Number	Gene Name	Target Sequence (5′-3′)	Reference
MIMAT0000062	let-7a-5p	UGAGGUAGUAGGUUGUAUAGUU	[32,33,34]
MIMAT0000069	miR-16-5p	UAGCAGCACGUAAAUAUUGGCG	[30,35,36,37]
MIMAT0004495	miR-22-5p	AGUUCUUCAGUGGCAAGCUUUA	[38,39]
MIMAT0000078	miR-23a-3p	AUCACAUUGCCAGGGAUUUCC	[37,40]
MIMAT0000080	miR-24-3p	UGGCUCAGUUCAGCAGGAACAG	[41]
MIMAT0000082	miR-26a-5p	UUCAAGUAAUCCAGGAUAGGCU	[30,33,40]
MIMAT0004503	miR-29a-5p	ACUGAUUUCUUUUGGUGUUCAG	[38]
MIMAT0000255	miR-34a-5p	UGGCAGUGUCUUAGCUGGUUGU	[41]
MIMAT0000099	miR-101-3p	UACAGUACUGUGAUAACUGAA	[39,40]
MIMAT0000101	miR-103a-3p	AGCAGCAUUGUACAGGGCUAUGA	[32]
MIMAT0000278	miR-221-3p	AGCUACAUUGUCUGCUGGGUUUC	[32,33]
MIMAT0004748	miR-423-5p	UGAGGGGCAGAGAGCGAGACUUU	[42]
MIMAT0003393	miR-425-5p	AAUGACACGAUCACUCCCGUUGA	[42]
MIMAT0003338	miR-660-5p	UACCCAUUGCAUAUCGGAGUUG	[38]
NR_004394.1	U6 snRNA	GUGCUCGCUUCGGCAGCACAUAUACUAAAAU UGGAACGATACAGAGAAGAUUAGCAUGGCCC CUGCGCAAGGAUGACACGCAAAUUCGUGAAG CGUUCCAUAUUUU	[43]
miRNA target			
MIMAT0004767	miR-193b-5p	CGGGGUUUUGAGGGCGAGAUGA	[44]

**Table 2:** Stability levels of candidate RGs in the different and merged experimental conditions.

**(A) CTRL**										
Ranking Order	Gene Name	Geomean	Genorm M-Value		Normfinder SV		BestKeeper SD		Delta CT SD	
1	miR-23a-3p	1.19	0.074	(1)	0.037	(1)	0.07	(2)	0.64	(1)
2	miR-425-5p	1.57	0.074	(1)	0.037	(2)	0.05	(1)	0.65	(3)
3	miR-660-5p	3.98	0.122	(3)	0.055	(3)	0.10	(3)	0.69	(7)
4	U6 snRNA	4.16	0.156	(4)	0.106	(5)	0.10	(4)	0.66	(5)
5	miR-16-5p	4.36	0.219	(5)	0.203	(6)	0.19	(6)	0.64	(2)
6	miR-423-5p	5.38	0.250	(7)	0.081	(4)	0.16	(5)	0.67	(6)
7	miR-24-3p	5.86	0.235	(6)	0.246	(7)	0.20	(7)	0.65	(4)
8	miR-26a-5p	8.00	0.303	(8)	0.501	(8)	0.38	(8)	0.77	(8)
9	miR-221-3p	9.00	0.402	(9)	0.935	(9)	0.65	(9)	1.02	(9)
10	miR-103a-3p	10.00	0.485	(10)	1.109	(10)	0.75	(10)	1.15	(10)
11	let-7a-5p	11.00	0.719	(11)	1.568	(11)	1.10	(11)	1.68	(11)
12	miR-34a-5p	12.00	0.940	(12)	2.006	(12)	1.41	(12)	2.05	(12)
**(B) OA-SF**										
Ranking Order	Gene Name	Geomean	Genorm M-Value		Normfinder SV		BestKeeper SD		Delta CT SD	
1	miR-221-3p	1.00	0.049	(1)	0.025	(1)	0.03	(1)	0.41	(1)
2	U6 snRNA	2.06	0.049	(1)	0.101	(3)	0.07	(2)	0.42	(3)
3	miR-16-5p	2.45	0.116	(3)	0.063	(2)	0.08	(3)	0.42	(2)
4	miR-24-3p	4.00	0.139	(4)	0.169	(4)	0.15	(4)	0.44	(4)
5	miR-660-5p	5.23	0.183	(5)	0.356	(6)	0.22	(5)	0.51	(5)
6	miR-26a-5p	5.96	0.289	(7)	0.277	(5)	0.27	(6)	0.52	(6)
7	miR-423-5p	6.74	0.233	(6)	0.51	(7)	0.32	(7)	0.61	(7)
8	miR-23a-3p	8.00	0.350	(8)	0.541	(8)	0.34	(8)	0.65	(8)
9	let-7a-5p	9.49	0.474	(10)	0.55	(9)	0.44	(10)	0.70	(9)
10	miR-425-5p	9.49	0.417	(9)	0.602	(10)	0.42	(9)	0.72	(10)
11	miR-34a-5p	11.00	0.528	(11)	0.677	(11)	0.47	(11)	0.77	(11)
12	miR-103a-3p	12.00	0.590	(12)	0.831	(12)	0.59	(12)	0.90	(12)
**(C) ALL**										
Ranking Order	Gene Name	Geomean	Genorm M-Value		Normfinder SV		BestKeeper SD		Delta CT SD	
1	miR-24-3p	1.00	0.135	(1)	0.213	(1)	0.18	(1)	0.64	(1)
2	miR-16-5p	1.68	0.135	(1)	0.263	(2)	0.20	(2)	0.66	(2)
3	miR-660-5p	3.22	0.300	(4)	0.291	(3)	0.20	(3)	0.70	(3)
4	miR-423-5p	4.47	0.368	(5)	0.296	(4)	0.24	(4)	0.70	(4)
5	miR-26a-5p	5.45	0.218	(3)	0.365	(7)	0.33	(7)	0.71	(6)
6	miR-23a-3p	5.48	0.408	(6)	0.306	(5)	0.28	(6)	0.70	(5)
7	miR-425-5p	5.86	0.433	(7)	0.354	(6)	0.24	(5)	0.74	(7)
8	miR-221-3p	8.00	0.484	(8)	0.643	(8)	0.42	(8)	0.84	(8)
9	U6 snRNA	9.24	0.557	(9)	0.766	(9)	0.71	(10)	0.97	(9)
10	miR-103a-3p	9.74	0.617	(10)	0.881	(10)	0.67	(9)	1.03	(10)
11	let-7a-5p	11.00	0.731	(11)	1.054	(11)	0.83	(11)	1.23	(11)
12	miR-34a-5p	12.00	0.876	(12)	1.521	(12)	1.12	(12)	1.60	(12)

In brackets () the position in the ranking of each different algorithm.

## Data Availability

Data are available in a publicly accessible repository: https://osf.io/wgc2n/?view_only=0281ce275cdf4c61ad90ab752799e42a (accessed on 10 December 2021).

## Supplementary Materials

The following supporting information can be downloaded at: https://www.mdpi.com/article/10.3390/biom12020316/s1, Table S1: C_RT_ values of positively amplified EV-miRNA RGs and OA-related miR-193b-5p.
