# Supplementary material for: Endogenous Controls for the Evaluation of Osteoarthritis-Related miRNAs in Extracellular Vesicles from Bone-Marrow-Derived Mesenchymal Stromal Cells and the Impact of Osteoarthritis Synovial Fluid
Source: Biomolecules. 2022 Feb 16;12(2):316. doi: 10.3390/biom12020316 (PMC8869367; doi:10.3390/biom12020316)
Supplement: Supplementary file 1 [file biomolecules-12-00316-s001.zip › Supplementary Table S1 - Biomolecules.pdf]

Table S1: C_{RT} values of positively amplified EV-miRNA RGs and OA-related miR-193b-5p

miRBase ID	C_{RT}	C_{RT}	C_{RT}	C_{RT}	C_{RT}	C_{RT}
	B11C	B15C	B16C	B11L	B15L	B16L
let-7a-5p	18.88	20.87	21.84	19.79	20.83	19.90
miR-16-5p	16.29	15.87	15.84	16.41	16.31	16.19
miR-23a-3p	22.09	22.09	22.25	21.44	21.66	22.31
miR-24-3p	12.49	12.07	11.99	12.51	12.16	12.20
miR-26a-5p	20.86	19.93	20.07	20.57	20.51	19.94
miR-34a-5p	15.75	17.06	19.58	19.19	18.60	17.84
miR-103a-3p	25.43	23.93	23.56	23.84	24.84	23.17
miR-221-3p	18.39	17.01	16.85	16.83	16.77	16.86
miR-423-5p	22.12	21.83	22.26	21.89	21.23	22.01
miR-425-5p	19.44	19.55	19.57	18.94	19.84	19.93
miR-660-5p	20.83	21.13	20.96	21.37	20.86	21.36
U6 snRNA	14.83	14.88	14.63	13.40	13.26	13.44
	B11C	B15C	B16C	B11L	B15L	B16L
miR-193b-5p	23.26	23.54	23.55	21.94	21.87	22.25
